# Current status and progress of the development of prostate cancer vaccines

**DOI:** 10.7150/jca.80803

**Published:** 2023-04-01

**Authors:** Jie Wang, Kaichen Zhou, Huihuang Zhu, Fukun Wei, Sai Ma, Yi Kan, Bingheng Li, Lijun Mao

**Affiliations:** 1 Department of Urology, the Affiliated Hospital of Xuzhou Medical University, Xuzhou 221000, China.; 2 Jiangsu Key Laboratory of Biological Cancer Therapy, Xuzhou Medical University, Xuzhou 221002, China.

**Keywords:** prostate cancer, cancer vaccine, tumor specific antigen, tumor associated antigen

## Abstract

At present, common treatments of prostate cancer mainly include surgery, radiotherapy, chemotherapy and hormone therapy. However, patients have high recurrence rate after treatment, and are prone to castration-resistant prostate cancer. Tumor vaccine is based on tumor specific antigen (TSA) and tumor associated antigen (TAA) to activate specific immune response of the body to cancer cells. With continuous maturity of tumor vaccine technology, different forms of prostate cancer vaccines have been developed, such as cellular vaccines, extracellular-based anti-tumor vaccines, polypeptide vaccines, and nucleic acid vaccines. In this review, we summarize current status and progress in the development of prostate cancer vaccines.

## Introduction

Prostate cancer is the second most common cancer among men in the world, and there are 1,414,259 new cases and 375,304 deaths worldwide in 2020. In China, with the acceleration of population aging, the growth of economic level and the improvement of detection methods, the incidence of prostate cancer is on the rise in recent years 1.

The mechanism of anticancer vaccines is the immune activation of specific tumor-associated antigens (TAA) or tumor-specific antigens (TSA), which can actively trigger the immune response by binding to costimulatory molecules localized on immune cells of cancer patients 2,3. Dendritic cells (DCs) are a kind of antigen presenting cells (APCs) that play an important role in antigen cross presentation 4. The discovery of antigen cross presentation has a far-reaching impact on the development of therapeutic tumor vaccines.

Prostate cancer has a large number of highly targeted TAA and TSA, such as PCA3, PAP, PSA, PSMA 5. Therefore, prostate cancer is an ideal candidate for cancer vaccine therapy, and most of these studies focus on metastatic castration-resistant prostate cancer (mCRPC) 6. Recently, several promising vaccines have been developed for prostate cancer treatment, such as Prostvac, Provenge (Sipuleucel-T) and individualized PPV (polypeptide vaccine). Provenge is the only cellular active immune product licensed by FDA to treat asymptomatic or mild symptomatic CRPC 7. Below we will provide further details of prostate cancer vaccines and summarize current status and progress in the development of prostate cancer vaccines.

## Dendritic cell vaccine

Dendritic cell vaccine is made of DCs loaded with tumor antigens. DC vaccines are inoculated into cancer patients to induce efficient anti-tumor response, including DC and tumor cell fusion vaccine, tumor antigen peptide or protein extract sensitized DC vaccine, gene modified DC vaccine, tumor cell extracellular sensitized DC vaccine 6. DCs are professional antigen presenting cells, so they can absorb, process and transmit antigen information.

Sapuleucel-T is currently the only active cellular immune product licensed by FDA to treat asymptomatic or mild symptomatic CRPC 7. Sipuleucel-T mainly includes peripheral blood monocytes activated by PAP and GM-CSF recombinant fusion protein *in vitro*, including antigen presenting cells. PAP is an enzyme secreted by the prostate and is highly expressed in metastatic prostate cancer and is the target of Sapuleucel-T 6. PA2024 antigen is a recombinant fusion protein containing PAP and GM-CSF, and GM-CSF receptors are widely expressed in APCs. The recombinant protein binds to the patient's APCs *in vitro* to make the APC display the antigen on its surface, and then the engineering APC is re-injected into the patient 7.

## Polypeptide vaccine

In the early 1980s, Lerner proposed determining the structure of natural antigens to synthesize peptides with both antigenicity and immunogenicity, and established a method for the development of synthetic peptide vaccines 8. TSA peptides delivered to the MHC (major histocompatibility complex) on the surface of APC (antigen presenting cell) are degraded in APC to form peptide-MHC-TCR complexes, which trigger the corresponding reaction of CTL (cytotoxic T lymphocyte). The advantage of peptide vaccine is that it is possible to combine various peptides obtained from different antigens into one carrier, and to construct corresponding synthetic antigenic peptides for complex discontinuous natural antigenic determinants 9. Therefore, prostate cancer polypeptide vaccines with many candidate TAA peptides have good application prospect.

GV1001 is an II-like telomerase polypeptide vaccine that can activate the immune system, leading to the activation of both CD4+ and CD8+T cell responses to recognize and kill tumor cells 10. Telomere terminal transferase activity is considered to be a common feature of all tumor cells. Therefore, GV1001 may be a universal tumor vaccine. At present, the vaccine has significantly improved the survival rate in a phase-adjusted I/II clinical trial in pancreatic cancer therapy, and a phase II clinical trial showed that the telomerase GV1001 polypeptide vaccine was well tolerated in the therapy of stage III NSCLC (non-small cell lung cancer) 11. However, there are no clinical trials of prostate cancer.

KRM-20 is a new tumor vaccine composed of 20 peptides to induce CTL against 12 different tumor-associated antigens highly expressed in prostate cancer tissues. A phase II study of KRM-20 showed that there was no evident difference in OS and PFS between the experimental group and the placebo group, but KRM-20 was effective in patients with chronic prostate cancer with lymphocyte ≥ 26% or PSA < 11.2 ng/ml 12.

Individualized polypeptide vaccine (personalized peptide vaccine, PPV) refers to the preparation of anti-tumor vaccine by selecting at most four peptides that match HLA-A1 (human leukocyte antigen A1) subtype according to the difference of individual genetic background 13. The advantage of PPV lies in the ability to bypass immune diversity and avoid immune tolerance. The results of phase I clinical trial showed that PPV was well tolerated and the level of PSA decreased 14. The results of the first randomized controlled phase II clinical trial of PPV in patients with CRPC showed that the overall survival (OS) of the low dose EMP group was significantly better than that of the standard dose EMP group 15. Another phase II randomized controlled clinical trial of 72 patients with CRPC showed that PFS and median OS in the PPV+ dexamethasone group were significantly higher than control group treated with dexamethasone alone 16. The patients selected in this study were in the early stage of CRPC, indicating that early CRPC patients can significantly improve PFS and OS with PPV treatment. In addition, the phase III clinical trial of PPV is being carried out, but the results have not been reported.

## DNA Vaccine

DNA vaccine is a closed circular DNA plasmid designed to encode antigens or epitopes of interest under the strong promoter of mammals 17. The mechanism of DNA vaccine is that naked plasmid DNA can induce the production of endogenous antigens and the presentation of MHC-I molecules. By changing the sequence of plasmid DNA, DNA vaccines can induce strong anti-tumor cellular immune response against tumor antigens 18. As a simple and effective antigen presentation method, DNA vaccine can have an impact on the treatment of prostate cancer. It is still a challenge to identify and select suitable tumor-specific antigens to cause minimal damage to normal cells.

PCaA-SEV (Prostate Canner Antigens-Synthetic Enhanced DNA Vaccine) is an enhanced DNA vaccine platform for the synthesis of multiple prostate cancer antigens. Candidate vaccines include PSCA, PAP, PCTA, STEAP1, PARM1 and PSP94. Studies have shown that cellular immunity to PCAA and versatility of antigen-specific T cells can be induced by PCAA-SEV. It is worth noting that mice immunized with PSP94 DNA vaccine showed the strongest cellular response. Similarly, candidate vaccines of PSCA, PCTA and PARM1 showed a strong cellular response against antigen. Compared with other candidate vaccines, splenocytes of mice immunized with STEAP1 and PAP-SEV showed a lower immune response. The enhanced delivery of these DNA vaccines mediated by the electroporation of plasmid (EP) can produce PCAA-specific CD8+T cells and increase their levels in tumor microenvironments, thereby improving the survival of mice carrying prostate cancer 19. Although it is often reported that EP can increase the immunogenicity of vaccines, the conversion of this technique into human trials is slightly 2-3 times higher than that of simple injection of naked DNA 20. The advantage of DNA vaccine is that it can combine candidate vaccines to attack multiple targets at the same time to inhibit tumor growth.

The monoclonal antibody against PSMA encoded by DNA is a new vaccine strategy that uses synthetic DNA plasmid to encode human anti-PSMA monoclonal antibody. The level of PSMA in prostate cancer cells is further increased. Studies have shown that the increased expression of PSMA is closely related to the progression of prostate cancer. PSMA is an attractive target for the development of anti-PSMA monoclonal antibodies for diagnostic and therapeutic purposes because it is a membrane protein 21. A study has shown that delivering PSMA-DMAb plasmids through EP can guide the production of strong levels of PSMA-specific human immunoglobulin *in vivo*. PSMA on the surface of human tumor cells can not only be recognized but also can be specifically bound by PSMA-DMAb 22. The synthesized PSMA-DMAb can bind to Fc receptors and mediate the ADCC effect, which is at least in part mediated by NK cells 23. This is the first report about the involvement of DNA vector of monoclonal antibody in host NK immune clearance and the use of delivery system based on DNA plasmid to guide the production of therapeutic mAb targeting tumor antigen PSMA *in vivo*.

PTVG-AR, MVI-118 is a DNA vaccine encoding the androgen receptor ligand binding domain, and is mainly tested in patients with mCSPC. In the preclinical model evaluation, it was found that AR expression increased in prostate tumor cells deprived of androgen, which made tumor cells more likely to be dissolved by AR specific CD8+T cells. The combination of AR targeted vaccine and androgen deprivation delayed tumor development in ovariectomized mice 24. An open-label, randomized, multi-agency phase 1 trial showed that 27 of 40 patients (68%) had no progress at 18 months, and there was no distinct difference in the time of castration resistance or first PSA rise between the study groups with or without GM-CSF adjuvant 25. Surprisingly, vaccine adjuvant GM-CSF did not induce better immune response, and preclinical studies of pTVG-AR vaccines in mice and rats have demonstrated the effect without additional adjuvants 26. In addition, it was found that immunization could stimulate T cells to respond to ARLBD, but could not cause antibody response, which was consistent with the results of preclinical studies in mice. It was not antibody response but DNA immunization promoted the production of CD4 and CD8T cells 26.

## RNA Vaccine

The most important RNA vaccine is the mRNA vaccine platform, which combines the immunological characteristics of live attenuated vaccine, the expression of endogenous antigens and the immunological characteristics of T cell induction and inactivated vaccine, such as determined composition and safety. RNA does not interact with the genome because of few cases of recombination between single-stranded RNA, and mRNA only contains the direct elements necessary to express the encoded protein, and mRNA does not need to cross the nuclear membrane, so it has safety advantages 27. MRNA binds to pattern recognition receptors, and messenger ribonucleic acid vaccines may be designed to be self-regulating, a feature missing from peptide and protein based vaccines 28.

The anti-tumor vaccine CV9103 contains four self-adjuvant mRNAs, which encode PSA, STEAP1, PSMA and PSCA 29. CV9103 encodes full-length antigens, which can induce immune responses against all epitopes contained in the target protein without being restricted by HLA. The existence of multiple antigens reduces the risk of tumor immune escape caused by the lack of expression of a single antigen 30. In a first-person I/IIA phase study of CV9103, 44 patients with CRPC were enrolled, of whom 12 were included in the first phase of the study to determine the safe dose of IIa; 32 patients entered the second phase, and Kaplan-Meier estimated the median OS of 36 metastatic CRPC patients treated with CV9103 at 31.4 months. It was observed that immune response and survival time had no significant correlation. The median OS was 29.3 months in all 44 patients. The median OS of subgroup patients with metastatic diseases was 31.4 months 29. Based on these results, CV9103 has good tolerance and immunogenicity. In addition, a phase IIb study of CV9104 began in 2012. CV9104 is an improved version of CV9103 vaccine that includes additional mRNA molecules that encode PAP and mucin 1 (MUC1) 31.

Adjuvant-pulsed mRNA vaccine nanoparticle (NP) includes an ovalbumin-coded mRNA, a palmitic acid-modified C16-R848 and a shell composed of lipid-polyethylene glycol. The combined transmission of C16-R848 adjuvant and OVA mRNA increases the presentation of TAA. The vaccine retains the adjuvant activity of C16-R848 and significantly increases the transfection rate of OVA messenger RNA in antigen presenting cells (> 95%) and subsequent presentation of MHC-I. Compared with the adjuvant-free mRNA vaccine NP, the mRNA vaccine NP regimen pulsed with C16-R848 adjuvant significantly improved the expansion of OVA-specific CD8+T cells *in vivo* and their invasion to the tumor bed, and induced a strong adaptive immune response 32. This approach resulted in effective anti-tumor immunity against allogeneic mouse lymphoma and prostate cancer models expressing OVA, which significantly prevented tumor growth when the vaccine was given before tumor implantation (84% less than the control) and after transplantation (60% less than the control) 32. In addition, an advantage of NP-mediated R848 over free R848 is to evade systemic cytokine responses, thereby reducing systemic toxicity 33.

MS2VLP-based PAP-GM-CSF mRNA vaccine is a recombinant phage MS2 virus-like particle (VLP) based on the interaction between 19-nucleotide RNA aptamer and MS2 phage coat protein, in which the target gene is packaged by MS2 capsid to protect the target RNA from nucleotide degradation, and then non-toxic and anti-ribonuclease MS2VLP gene vaccine can be easily produced by using recombinant protein technology 34. The Th1/Th2 response was balanced without upregulating CD41 regulatory T cells. Ganglioside GD1a is highly expressed in CRPC cells. HVJ-E selectively binds to it, resulting in apoptosis of prostate cancer cells 35. The capsid of MS2 can be modified by inserting peptides to target specific cells, and this vaccine is expected to be developed jointly with polypeptide vaccines.

## Virus vaccine

As a new type of viral preparation for the treatment of CRPC, oncolytic adenovirus has the advantages of high selectivity and low cytotoxicity 36. According to the oncolytic characteristics of Ad5, Ad5 is divided into two vectors: CRAd vector (conditional replication adenovirus) and RDAd vector (replication defective adenovirus). Conditional replication adenovirus vector can only proliferate and destroy cancer cells, but cannot replicate in normal cells, which is also called oncolytic adenovirus. Replication defective adenovirus vector have no intracellular replication, but can carry remedial genes 37. The insertion of foreign genes and deletion of viral cytoskeleton genes can enhance the oncolytic ability of adenoviruses. For example, the oncolytic mutant Ad deleted without E1B19K and E1ACR2 can induce apoptosis of prostate cancer cells 38. In addition, the addition of prostate-specific promoters / enhancers only leads to viral replication and foreign gene expression in prostate cancer cells 39. PSA, PSMA, PB and PCA3/DD3 promoters have been used in adenovirus-mediated gene therapy of prostate cancer. Ad/PSAP-GV16- β G vector combined with prodrug DOX-GA_3 can kill LNCaP cell transplanted tumor in nude mice. Ad-PSMA (Emurp)-CD vector based on PSMA promoter drives the expression of cytosine deaminase, and effectively kills PSMA-producing CL-1 transplanted tumor with combined use of prodrug 5-flucytosine 40. Ad vector based on PB promoter may target AR positive PCa 41. Hao et al. designed OncoAd.mK5.DD3 vector that effectively inhibited PCa 42.

Sendai virus is a kind of virus with cell fusion activity but loss of replication ability after UV inactivation. HVJ-E (HVJ envelope) retains the ability of cell fusion 43. HVJ-E selectively binds to Ganglioside GD1a and causes the apoptosis of PCa cells. In a phase I / II trial, 7 patients were selected and 6 patients were treated with HVJ-E. The PSA effective rate of HUJ-E treatment was 16.6%, and the adverse reactions were mild. The results showed that the level of PSA decreased completely in patients with metastatic prostate cancer 44. In another phase I dose increment study, the safety and efficacy of higher dose HVJ-E (GEN0101) were tested. The inhibitory effect on the increase of PSA with high dose was stronger than that in the low one. The NK cell activity was enhanced in 2 cases with low dose and 5 cases with high dose 45. The results showed that intratumoral and subcutaneous GEN0101 injection was well tolerated.

Prostvac is a tumor vaccine based on recombinant poxvirus vector, which consists of two components, one is recombinant vaccinia virus, which can stimulate the initial immune response, and the other is fowlpox virus, which is used to enhance immune response 46. Phase I clinical trials confirmed the safety and immune activity of Prostvac 47. In a phase II clinical trial, 82 patients were enrolled in the Prostvac treatment group and 40 patients in the control group. Three years after treatment, the mortality in the Prostvac group (69.5%) was significantly lower than that in the control group (82.5%). The average survival time of metastatic CRPC patients treated with Prostvac was significantly prolonged (25.1 months vs. 16.6 months) 48.

## The combination of prostate cancer vaccines and other treatments

Although prostate cancer vaccines show promise, it should be emphasized that the treatment of prostate cancer should be a combination of multiple treatment methods, such as immune checkpoint inhibitors (ICI), CART cells, prostate cancer vaccines, hormone therapy, radiotherapy and chemotherapy. Targeting the immune system may be a promising treatment for prostate cancer in the future. The combination of PD-1/PD-L1 blockers and other treatments has shown good results for prostate cancer 49. Anti-PD-1/PD-L1 combined with other drugs showed effective anti-tumor effects, including androgen deprivation, anti-tumor vaccine, radiotherapy and chemotherapy 50.

Androgen deprivation therapy (ADT) can induce T cells to activate prostate antigen and temporarily reduce the tolerance of T cells. A synergistic relationship between ADT and immunotherapy has been proposed 51. So far FDA has authorized the use of ipilimumab monoclonal antibody as cancer immunotherapy. Current clinical trials of mCRPC use a mixture of immune checkpoint inhibitors as an alternative. For example, CHECKMATE650, a phase II clinical study, began to examine the efficacy of a combination of ipilimumab and nivolumab in patients with mCRPC who are resistant to androgen receptor (AR) targeted therapy 52.

Recent studies showed better efficacy of combined immunotherapy and chemoradiotherapy for cancer treatment 53,54. Several clinical trial in mCRPC patients showed the benefits of combined chemotherapy and immunotherapy for prostate cancer therapy 55,56. After vaccination with tumor antigen-specific DNA vaccine, the expression of PD-L1 on circulating tumor cells (CTCs) increased, which was related to the development of T cell immunity and longer progression-free survival. These findings provide the support for the combination of anticancer vaccines and PD-1 blocking antibodies in the treatment of prostate cancer 57. In TRAMP mice in advanced stage of prostate cancer, VLP vaccine alone or in combination with anti-PD1 antibody could significantly reduce the tumor burden by using novel virus-like particles (VLP) vaccine, anti-PD1 antibody or combined immunotherapy 58. Continuous injection of anti-PD-1 and anti-TIM-3 antibodies further improved the therapeutic effect of anchored granulocyte-macrophage colony-stimulating factor (GM-CSF) vaccine. Tumor regression was found in more than 60% of mice. This triple therapy can increase specific cytotoxic activity, proliferation and secretion of CD8+TIL, and reduce the production of tumor-promoting cytokines. These results suggest that triple therapy can obtain effective anti-tumor immune response in patients with prostate cancer 59.

In addition, other chemotherapeutic drugs have been shown to improve the anticancer effect of PCa when CTLA-4 and PD1/PD-L1 are inhibited. A study reports that hip-T vaccine can increase immune penetration of prostate cancer and create a favorable environment for PD-1/PD-L1 blocking, suggesting that the combination of hip-T vaccine and PD-1/PD-L1 blocking may be a promising immunotherapy strategy 60.

## Conclusion

**Figure 1:**
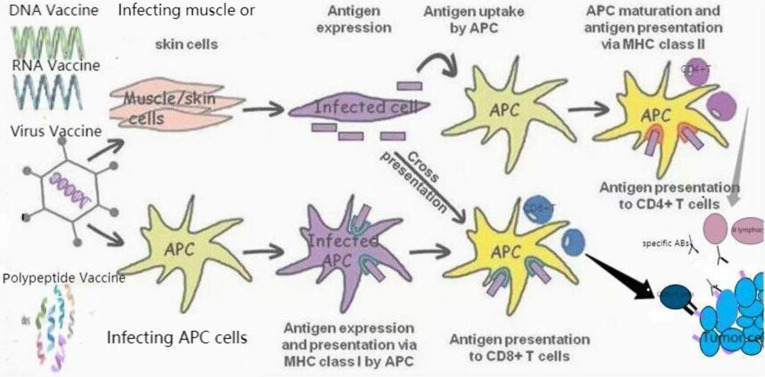
Therapeutic vaccines for prostate cancer. TAAs can be transmitted through different vaccine forms (RNA or DNA vaccines encoding TAAs, polypeptide vaccines, viral vaccines). APC induces specific cytotoxicity to tumor cells expressing TAA through the interaction of MHC class I with CD8+T cells. CD4+T cells were activated by the second type of MHC, APC. In addition, they also induce the activation of B lymphocytes to produce specific antibodies (Abs) against tumor cells expressed by TAA.

In recent years, prostate cancer vaccine has received more attention (Figure 1). In this review, we summarize current status of the development of prostate cancer vaccines and highlight some emerging vaccine strategies (Table 1). The combination of dendritic cell vaccine, peptide vaccine and nucleic acid vaccine may be a promising strategy in the future. In addition, simple tumor vaccine therapy is difficult to achieve high treatment efficacy, and the combination with chemotherapy, radiotherapy and surgery may be necessary as discussed in previous section. It can be predicted that treatment strategy based on the combination of a variety of tumor vaccines and traditional treatment methods supplemented with immune adjuvants will become a major trend of anti-tumor therapy for prostate cancer.

## Figures and Tables

**Table 1:** Prostate cancer vaccines under development.

Vaccines	Specific Antigens	Immune System Effectors	Clinical Outcomes	Side Effects
Sapuleucel-T	PAP	Antigen presenting cells	FDA licensed cellular active immune products for asymptomatic or mild symptomatic CRPC	chills (53.1%), fever (31.3%), muscle pain (11.8%) and headache (18.1%).
GV1001	Telomerase polypeptide	CD4+ and CD8+ T cells	There is no clinical trial in prostate cancer.	/
KRM-20	20 peptides	CTL	Effective for chronic prostate cancer patients with lymphocyte ≥ 26% or PSA < 11.2 ng/ml	No serious adverse reactions
PPvs	Up to four peptides that match human leukocyte antigen A1 (HLA-A1) are selected	CTL	Early CRPC patients can significantly improve PFS and OS	Good tolerance
PCaA-SEV	STEAP1, PAP, PARM1, PCTA, PSCA	T cell	Mice immunized with PSP94DNA vaccine showed the strongest cellular response.	/
PSMA-DMAb	PSMA	NK cells, T cells	This is the first report about the involvement of DNA vector monoclonal antibody in host NK immune clearance and the use of DNA plasmid-based delivery system to guide the production of therapeutic mAb targeting related oncology target PSMA *in vivo*.	No serious adverse reactions
pTVG-AR, MVI-118	Androgen receptor	CD8+T cells	Patients treated with vaccine had significantly longer first PSA rise	/
CV9103	PSA, PSMA, PSCA, STEAP1	T cells	The median OS of all 44 patients was 29.3 months. In the subgroup of patients with metastatic diseases median OS was 31.4 months.	Good tolerance and immunogenicity
Adjuvant-pulsed mRNA vaccine nanoparticle	Adjuvant-pulsed mRNA vaccine NP	CD8+T cells	tumor growth was significantly inhibited.	Low systemic toxicity
MS2 VLP-based PAP-GM-CSF mRNA vaccine	TSA	B cells and T cells	The Th1/Th2 response was balanced without upregulating CD41 regulatory T cells, and protected C57BL/6 mice from prostate cancer and delayed tumor growth.	No serious adverse reactions
HVJ envelope (HVJ-E)	Sendai virus	T cells	In a single-arm, open-label, single-center, phase I/II HVJ-E study, the PSA response rate of HVJ-E was 16.6%.	Mild adverse reactions
Prostvac	PSA, Cowpox virus, Fowlpox virus	T cells	The average survival time of patients with metastatic CRPC was significantly prolonged after treatment	Mild side effects (fever and injection site reaction)
